# Rupture of Renal Artery Aneurysm in a Patient with Granulomatosis with Polyangiitis

**DOI:** 10.31662/jmaj.2021-0027

**Published:** 2021-09-01

**Authors:** Kyosuke Matsunaga, Junya Tsurukiri, Takahisa Kato, Hiroki Suenaga, Naruaki Otake, Jushi Numata, Tadasu Kojima, Takashi Oda

**Affiliations:** 1 Department of Emergency and Critical Care Medicine, Tokyo Medical University Hachioji Medical Center, Tokyo, Japan; 2 Department of Nephrology and Blood Purification, Kidney Disease Center, Tokyo Medical University Hachioji Medical Center, Tokyo, Japan

**Keywords:** interventional radiology, embolization, hemorrhage, granulomatosis with polyangiitis, renal artery aneurysm

## Abstract

Granulomatosis with polyangiitis (GPA) is the systemic vasculitis affecting predominantly small vessels, but vasculitis of medium size artery can be associated. We treated a patient with GPA who had hemorrhagic instability because of a rupture of an aneurysm in the branch of the renal artery; the patient underwent arterial embolization (AE), and hemostasis was successfully achieved. Literature reviews were conducted on the basis of the data available on PubMed, and seven published reports of eight cases with renal artery aneurysms were identified. We concluded that emergency physicians should be aware of the existence of renal artery aneurysms associated with GPA. AE should be considered as one of the treatment choices whenever renal bleeding takes place.

## Introduction

Granulomatosis with polyangiitis (GPA), microscopic polyangiitis, and eosinophilic granulomatosis with polyangiitis are three major clinical entities of small vessel vasculitis recognized among antineutrophil cytoplasmic antibody (ANCA)-associated vasculitis. GPA is more often, but not exclusively, associated with proteinase 3 (PR3)-ANCA^(1)^. We treated a patient diagnosed with GPA who became hemorrhagic shock because of the rupture of renal artery aneurysm.

## Case Report

**Figure 1.**
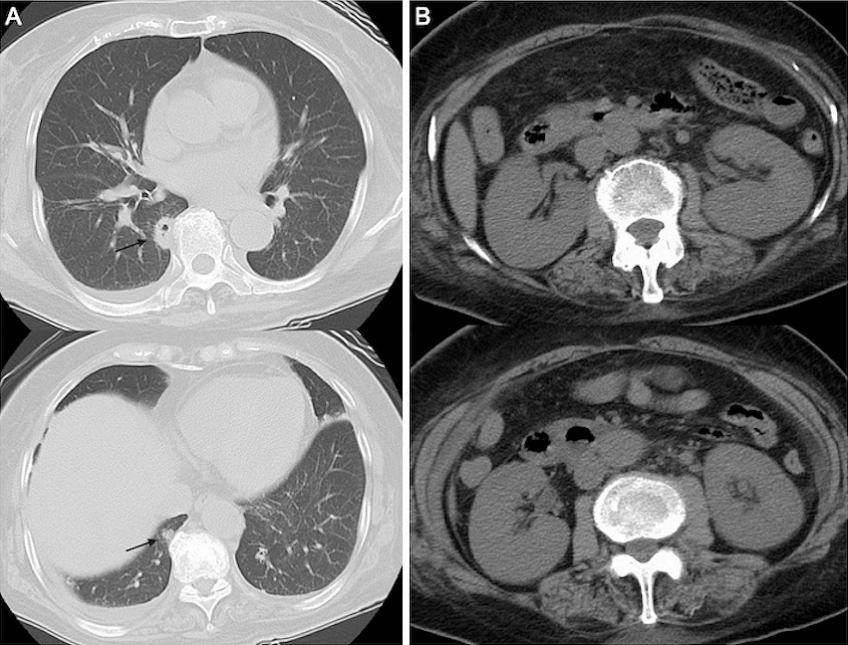
(A) Computed tomography (CT) of the chest revealed multiple small nodular infiltrates with cavities (arrow). (B) Abdominal CT revealed the enlargement of both the kidneys.

Seven days earlier, a 75-year-old woman had been admitted to another hospital for fever of unknown origin and dry cough for 12 days. During hospitalization, deterioration of renal function with an elevation of serum creatinine level (1.25-5.53 mg/dL) occurred, and there was no improvement of fever and cough; thus, she was transferred to our hospital. She had a medical history of hypertension and arrhythmia. Physical examination revealed the following: consciousness, alert; blood pressure, 104/61 mmHg; heart rate, 85 beats/min; respiratory rate, 24 breaths/min; and body temperature, 37.4℃. Laboratory test results are listed in Table 1. Computed tomography (CT) of the chest revealed multiple small nodular infiltrates with cavities in both lungs (Figure 1A). Abdominal CT showed enlargement of both kidneys (Figure 1B). The clinical diagnosis of GPA was made on the basis of the upper airway and kidney symptoms along with positive PR3-ANCA. Thus, we administered intravenous methylprednisolone at 1 g/day for 3 days and subsequently at 40 mg/day.

**Table 1.** Laboratory Test Performed at Our Hospital.

*Variables*	Results (reference range)
*Blood test*	
1) C reactive protein	18.93 mg/dL (<0.14 mg/dL)
2) D-dimer	34.08 μg/mL (<0.14 mg/dL)
3) erythrocyte sedimentation rate	49 mm (<20mm)
4) creatinine	6.07 mg/dL (0.46-0.79 mg/dL)
5) PR3-ANCA	150.50 U/mL (<3.5 U/mL)
6) MPO-ANCA	0.00 (<3.5 U/mL)
7) intact parathyroid hormone	165.80 pg/mL (10.0-65.0 pg/mL)
*Urine test*	
1) Protein	200 mg/dL
2) Creatinine	64 mg/dL
3) Red blood cells	>100 /HPF (<4 /HPF)
4) Granular casts	positive (2+)

ANCA, anti-neutrophil cytoplasmic antibody; MPO, myeloperoxidase; and PR3, proteinase 3 Urinalysis showed a protein level of 3.47 g/gCr with hematuria of more than 100 RBCs/highpower field, and with 2+ granular casts.

**Figure 2.**
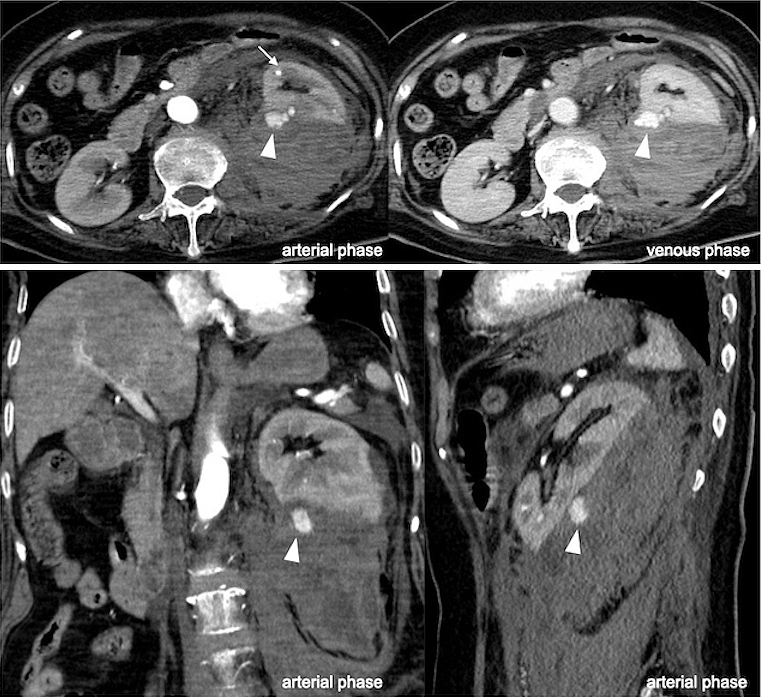
Images of computed tomography with abdominal hematoma. Contrast medium extravasation from the kidney (arrowhead) and aneurysm formation (arrow).

Ten days later, the patient complained of abdominal pain. She became hemodynamically unstable. Hemoglobin, hematocrit, and lactate levels were 9.1 g/dL, 27%, and 79 mg/dL (range, 4-16 mg/dL), respectively. Contrast-enhanced CT of the abdomen exposed a noteworthy retroperitoneal hematoma with contrast medium extravasation (CMEV) (Figure 2). The serum creatinine level at the time was improved to under 3.0 mg/dL. Therefore, we decided to perform arterial embolization (AE) to achieve hemostasis.

**Figure 3.**
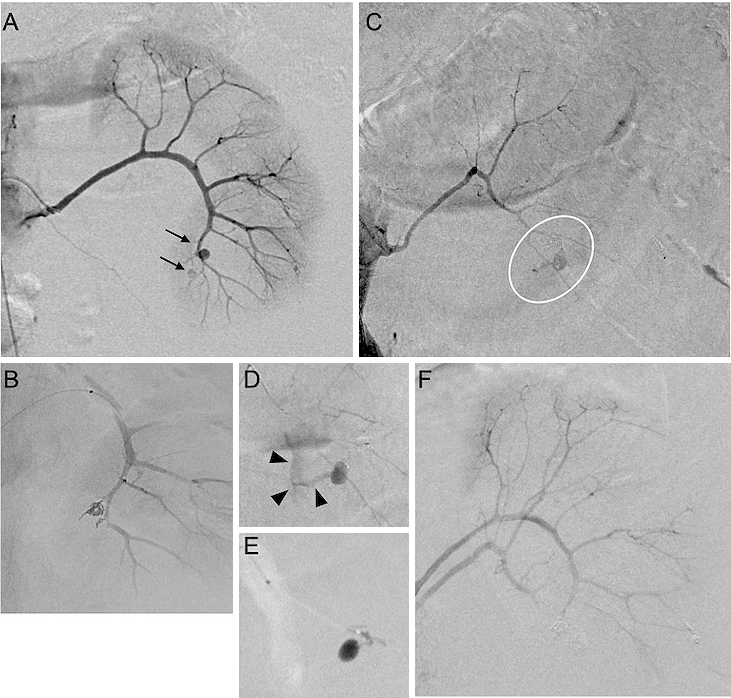
(A) Angiography revealed the aneurysms (arrow) from the anteroinferior segmental artery of the kidney. (B) Coil embolization. (C and D) Angiography showing the extravasation of contrast medium from the posterior segmental artery of the kidney. (E) Injection of N-butyl cyanoacrylate. (F) Completion of hemostasis.

Under general anesthesia, angiography revealed multiple aneurysms of the left renal segmental arteries. Initially, we initiated superselective AE using microcoils into the aneurysms arising from the branches of the left anteroinferior segmental artery (Figure 3A and 3B). Subsequently, we applied selective catheterization to the left posterior segmental artery aneurysm, and then, angiography revealed CMEV (Figure 3C and 3D). AE was undertaken for the bleeding vessel, and hemostasis had been achieved (Figure 3E and 3F). A total of 1,680 mL of red blood cells and 1,200 mL of fresh frozen plasma were given within 24 h.

The administration of corticosteroids was continued, and its dosage was reduced by 50% after 31 days. Rituximab was initiated as induction therapy for GPA after 33 days. The patient was discharged from the hospital after 42 days without any complications and continues to remain symptom-free 6 months after AE.

## Discussion

GPA is a type of primary systemic ANCA-associated vasculitis. This rare disorder has two distinctive histopathological features: necrotizing small and medium vessel vasculitis and granulomatous inflammation. Although the lungs, upper airway, ears, nose, and sinuses are usually affected in the early phase, the kidney can be severely damaged by necrotizing vasculitis in the advanced stage.

A literature search using PubMed that included the terms “aneurysm” and “granulomatosis with polyangiitis” or “Wegener’s granulomatosis” was conducted. To date, nine published reports in the English language involving 10 GPA patients who had renal artery aneurysms were identified (Table 2) ^(2), (3), (4), (5), (6), (7), (8), (9), (10)^. Among them, four patients experienced rupture of renal artery aneurysms after the diagnosis of GPA and became hemorrhagic shock. Three of the four patients had positive PR3-ANCA. Two of the four cases were treated by AE (gelatin or coil), one patient received conservative therapy, and one patient underwent nephrectomy but died because of renal failure. Hence, nephrectomy or carbon dioxide angiography, which can replace iodinated contrast, are alternative approaches for hemostasis, but there may be technical difficulties in using them to treat patients with hemorrhagic shock.

**Table 2.** Review of the Published Literatures on Renal Artery Aneurysms in Patients with Granulomatosis with Polyangiitis.

Author, Year	No. of patients	Age	Sex	Diagnostic tool for GPA	Aneurysmal symptoms	Hemodynamics	Diagnostic tool for aneurysms	Duration between aneurysm and GPA diagnosis	Involved artery	Rupture artery aneurysm	Treatment	Outcome
Baker, 1978 ^(2)^	1	24	male	Biopsy	Rt. flank pain, shock	Unstable	Angiography	2 days	Renal	Yes (renal)	AE (gelfoam)	Recovery
Moutsopoulos, 1983 ^(3)^	2	30	male	Biopsy	NA	Stable	Angiography	4 months	Renal	No	Immunosupression	NA
		53	female	Clinical featues	NA	Stable	Angiography	1 month	Renal	No	Immunosupression	NA
Senf, 2003 ^(4)^	1	35	male	Biopsy, PR3-ANCA	Lt. flank pain, shock	Unstable	CT	24 days	Hepatic, renal, splanchnic	Yes (hepatic)	Immunosupression	Recovery
Arlet, 2007 ^(5)^	1	29	male	Biopsy, PR3-ANCA	Abdominal pain, vomitting	Stable	CT	5 years	PD, Hepatic, renal	No	AE (coil, for hepatic artery)	Recovery
Carron, 2011 ^(6)^	1	79	male	Clinical features, PR3-ANCA	Shock due to colon hematoma	Unstable	Angiography	4 weeks	Renal	No	Immunosupression	Recovery
Boersma, 2013 ^(7)^	1	51	female	PR3-ANCA	Lower abdominal and flank pain	Unstable	Angiography	15 years	Renal	Yes (renal)	AE	Recovery
Kim, 2017 ^(8)^	1	71	male	Biopsy, PR3-ANCA	none	Stable	CT	concomitant	Renal	No	Immunosupression	Recovery
Mahmudpour, 2018 ^(9)^	1	60	male	PR3-ANCA	Rt. flank pain, shock	Unstable	CT	NA	Renal	Yes (renal)	Nephrectomy	Died due to renal failure
Skonieczny, 2019 ^(10)^	1	50	male	Clinical features, PR3-ANCA	Rt. flank pain, shock	Unstable	CT	15 days	Renal	Yes (renal)	AE (coil), immunosupression	Died due to infection
Present case	1	75	female	Clinical features, PR3-ANCA	Abdominal pain, shock	Unstable	CT	20 days	Renal	Yes (renal)	AE (coil), immunosupression	Recovery

AE, arterial embolization; CT, computed tomography; GPA, gramulomatosis with polyangitis; Lt, left; NA, no available; PD, pancreatic-duodena; PR3-ANCA, proteinase3-anti-neutrophil cytoplasmic antibodies; and Rt, right

Aneurysms are sometimes observed in polyarteritis nodosa, a necrotizing arteritis of medium or small arteries without glomerulonephritis or vasculitis, and are not associated with ANCAs^(1)^. The ANCA that appeared in the blood is usually helpful for most people who have active GPA but are not specific for the diagnosis of the condition. Although PR3-ANCA is most common in America and Europe, myeloperoxidase-ANCA has been most often detected (54.6%) in Japan.

We continued the administration of corticosteroid for reducing the inflammation in the blood vessels combined with rituximab to control the immunologic abnormality. No further formation of renal artery aneurysms has been observed for 6 months. In conclusion, physicians should be aware of the existence of renal artery aneurysm associated with GPA, and AE could be the treatment of choice whenever renal bleeding occurs.

## Article Information

### Conflicts of Interest

The authors declare that there are no conflicts of interest.

### Acknowledgement

The authors would like to thank Enago (www.enago.jp) for the English language review.

### Author Contribution

Conceived and designed the experiments: MK, TJ

Contributed to interpretation of data: KT, SH, ON, NJ, KT

Approved the final version to be submitted: OT

### Informed Consent

Written informed consent was obtained from the patient for publication of this case report and any accompanying images.
